# The Correlation between Serum Levels of Anti-*Toxoplasma gondii* Antibodies and the Risk of Diabetes

**Published:** 2018

**Authors:** Maryam KHALILI, Mahmoud MAHAMI-OSKOUEI, Abbas SHAHBAZI, Abdolrasoul SAFAIYAN, Nader MOHAMMADZADEH-GHESHLAGHI, Leyla MAHAMI-OSKOUEI

**Affiliations:** 1. Immunology Research Center, Tabriz University of Medical Sciences, Tabriz, Iran; 2. Dept. of Parasitology and Mycology, Faculty of Medicine, Tabriz University of Medical Sciences, Tabriz, Iran; 3. Road Traffic Injury Research Center, Tabriz University of Medical Sciences, Tabriz, Iran; 4. Dept. of Biostatistics and Epidemiology, Faculty of Health, Tabriz University of Medical Sciences, Tabriz, Iran; 5. Central Medical Laboratory of East Azerbaijan Province, Tabriz University of Medical Sciences, Tabriz, Iran

**Keywords:** Diabetes mellitus, *Toxoplasma gondii*, Chemiluminescence, Immunoassay

## Abstract

**Background:**

This study investigated the presence of specific antibodies against *Toxoplasma gondii* infection among people with diabetes (type I and II) in comparison with control group.

**Methods:**

Overall 300 serum samples were collected equally from three groups including patients with type I and type II diabetes and non-diabetic healthy control that referred to Tabriz Central Laboratory in northwest Iran during July to Sep 2015. The level of specific IgG and IgM antibodies against *T. gondii* were measured using the chemiluminescence immunoassay (CLIA) method. Chi-square and One-Way ANCOVA were used for data analysis.

**Results:**

Overall, 300 samples from diabetic patients (type I and type II) and control group were examined and results showed 3, 8 and 2 cases were seropositive for anti-*T. gondii* IgM respectively. Anti-*T. gondii* IgG seropositivity in type I and type II diabetes and control groups were 69%, 63% and 59% respectively. We did not observe any statistical differences among all studied groups in terms of toxoplasmosis. There was no statistically significant relationship between all variables and seropositivity for anti-*T. gondii* antibodies in type I and II diabetes and non-diabetic groups.

**Conclusion:**

Although there was no statistically significant relationship between diabetes and toxoplasmosis further investigations especially experimental studies using animal models are needed. Furthermore, these findings would not be contrary to the need for healthcare in order to the prevention of infectious disease in diabetic patients.

## Introduction

*Toxoplasma gondii* is an obligate intracellular protozoan warm-blooded vertebrates and causes toxoplasmosis in humans (1). There are several ways to *T. gondii* infection, including consuming oocysts by food or water and tissue cysts from undercooked meat (2, 3). Humans are also infected by transmission from a pregnant woman that caused congenital toxoplasmosis (4, 5). *T. gondii* infects at least one-third of the population around the world (6, 7). Studies in different regions in Iran indicate the seroprevalence of *T. gondii* infection at 39.3% of general population (8). In most individuals acute infection with *T. gondii* is asymptomatic. Symptomatic acquired infections are usually associated with reticular cell hyperplasia and lymphadenopathy (9). Toxoplasmosis can also cause severe disease in immunocompromised patients such as HIV positive or patients treated with immunosuppressive drugs (10, 11).

Diabetes mellitus (DM) is one of the health-threatening concomitant diseases in the world. 285 million adults (6.4%) worldwide had diabetes in 2010 and the number of patients will increase to 439 million (7.7%) by 2030 (12). Diabetes is classified as chronic diseases that occur when the insulin production in the pancreas is disrupted or the cells cannot the ability to use the insulin (13). The tachyzoites of *T. gondii* rapidly metabolizes glucose via glycolysis. Immune responses in the host cells, maintaining regular metabolic, and development of disturbances occurs due to increasing requirements for glutamine and thus the risk of susceptibility to various infections increases in diabetes (14, 15).

In a systematic review analyzed the results of seven types of research has shown conflicting results regarding the relationship between toxoplasmosis and diabetes. Further studies should be considered to find out more about this association (16).

The present study was aimed to investigate the presence of specific antibodies against *T. gondii* infection among people with diabetes (type I and II) in comparison with the control group using the chemiluminescence immunoassay (CLIA) method in northwest Iran.

## Materials and Methods

In this case-control study, 200 cases including 100 patients with type I diabetes and 100 patients with type II diabetes and also 100 non-diabetic individuals as a control group randomly were selected from patients having referred to Tabriz Central Laboratory in northwest Iran during July to September 2015.

Inclusion criteria in diabetic groups were based on history (including family history and insulin therapy or received other drugs which reduce blood sugar), fasting blood sugar (more than 110 mg/dl) and HbA1c level (more than 6 percent). Control group were non-diabetic patients selected among those attending and matched with patient groups for age and gender. Patients with other metabolic disorders, immunocompromised and receiving immunosuppressive drugs were excluded from the present study.

After filling the questionnaire including age, gender, residency, education, contact with cat or cat keeping and eating raw animal products and also completing informed consent forms, 3 ml of blood were taken from each case and control groups. The samples were centrifuged and the sera were kept at −20 °C. The level of specific IgG and IgM antibodies against *T. gondii* were measured using the chemiluminescence immunoassay (CLIA) method by commercially available kit (Diasorin, Italy). More than 8.8 and 8 international units (IU)/ml were considered to be a positive value for IgG and IgM, respectively.

This study was approved by Ethics Committee of Tabriz University of Medical Sciences (TBZMED.REC.1394.404).

Data were analyzed using SPSS software (ver. 22 (Chicago, IL, USA). Chi-square and Fisher's exact tests were used to compare the seroprevalence values. One-Way ANCOVA was used for evaluating the relations between quantitative IgG and IgM with some important variables as the predictors by controlling the confounding variables. *P*<0.05 was considered as the level of significance.

## Results

Totally, 300 samples from diabetic patients (type I and type II) and control group were collected and serologic results showed 3, 8 and 2 cases were seropositive for anti- *T. gondii* IgM respectively. Our study showed that 42.3% of studied people were in 35–45 age groups. There was no statistically significant relationship between age, gender, education, job, residency and eating raw animal products and seropositivity for anti- *T. gondii* IgM in type I and II diabetes and non-diabetic groups (Table 1). We also evaluate the results of measured anti- *T. gondii* IgG titers. Seropositive cases in type I and type II diabetes and control groups were 69, 63 and 59 persons respectively. We could not find a statistically significant relation between all variables and seropositivity for anti- *T. gondii* IgG in type I and II diabetes and non-diabetic groups (Table 2). Although anti- *T. gondii* IgM and anti- *T. gondii* IgG seropositivity rate were different in men and women but this difference was not statistically significant between the studied groups. We did not observe any statistical differences among all studied groups in terms of toxoplasmosis.

**Table 1:** Comparison of demographic characteristics and risk factors in *T. gondii* IgM positive cases among diabetic and non-diabetic groups

***Factor***	***Group (Total)***			***Group (IgM Positive)***			***P-value***
	***Control (%)***	***Type I (%)***	***Type II (%)***	***Control (%)***	***Type I (%)***	***Type II (%)***	
***Age***							
<35 yr	38(40.43)	32(34.04)	24(25.53)	1(2.63)	2(6.25)	3(12.5)	0.739
35–45	32(25.2)	40(31.5)	55(43.3)	1(3.12)	0(0)	4(7.27)	
>45 yr	30(37.97)	28(35.45)	21(26.58)	0(0)	1(3.57)	1(4.76)	
***Gender***							
Male	50(33.33)	50(33.33)	50(33.33)	1(2)	1(2)	1(2)	0.713
Female	50(33.33)	50(33.33)	50(33.33)	1(2)	2(4)	7(14)	
***Education***							
School and below	17(29.82)	23(40.36)	17(29.82)	1(5.88)	1(4.33)	0(0)	0.284
Under-graduate	32(26.01)	42(34.15)	49(39.84)	0(0)	1(2.38)	5(10.2)	
Post-graduate	51(42.5)	35(29.17)	34(28.33)	1(1.96)	1(2.85)	3(8.82)	
***Job***							
Employee	25(29.41)	28(32.94)	32(37.65)	0(0)	1(3.57)	2(6.25)	0.230
Self-employed	30(37.5)	25(31.25)	25(31.25)	1(3.33)	0(0)	0(0)	
Housekeeper	45(33.33)	47(34.82)	43(31.85)	1(2.22)	2(4.25)	6(13.95)	
***Residency***							
H ^*^	28(29.79)	25(26.59)	41(43.62)	1(3.57)	1(4)	3(7.31)	0.809
M ^**^	39(41.05)	31(32.63)	25(26.32)	1(2.56)	0(0)	2(8)	
L ^***^	33(29.73)	44(39.63)	34(30.64)	0(0)	2(4.54)	3(8.82)	
***Eating raw animal products***							
Yes	42(30.66)	58(42.34)	37(27)	2(4.76)	2(3.44)	5(13.51)	
NO	58(35.58)	42(25.77)	63(38.65)	0(0)	1(2.38)	3(4.76)	
							0.762

* H: Living in high-income areas;

** M: Living in middle-income areas;

*** L: Living in low-income areas

**Table 2:** Comparison of demographic characteristics and risk factors in *T. gondii* IgG positive cases among diabetic and non-diabetic groups

***Factor***	***Group (Total)***			***Group (IgG Positive)***			***P-value***
	***Control (%)***	***Type I (%)***	***Type II (%)***	***Control (%)***	***Type I (%)***	***Type II (%)***	
***Age***							
<35 yr	38(40.43)	32(34.04)	24(25.53)	18(47.36)	13(40.62)	15(62.5)	
35–45	32(25.2)	40(31.5)	55(43.3)	24(75)	31(77.5)	32(58.18)	0.094
>45 yr	30(37.97)	28(35.45)	21(26.58)	17(56.66)	25(89.28)	16(76.19)	
***Gender***							
Male	50(33.33)	50(33.33)	50(33.33)	34(68)	36(72)	34(68)	0.437
Female	50(33.33)	50(33.33)	50(33.33)	25(50)	33(66)	29(58)	
***Education***							0.540
School and below	17(29.82)	23(40.36)	17(29.82)	10(58.82)	16(69.56)	11(64.7)	
Under-graduate	32(26.01)	42(34.15)	49(39.84)	22(68.75)	31(73.8)	31(63.26)	
Post-graduate	51(42.5)	35(29.17)	34(28.33)	27(52.94)	22(62.85)	21(61.76)	
***Job***							0.834
Employee	25(29.41)	28(32.94)	32(37.65)	14(56)	18(64.28)	22(68.75)	
Self-employed	30(37.5)	25(31.25)	25(31.25)	22(73.33)	20(80)	16(64)	
Housekeeper	45(33.33)	47(34.82)	43(31.85)	23(51.11)	31(65.95)	25(58.13)	
***Residency***							
H ^*^	28(29.79)	25(26.59)	41(43.62)	15(53.57)	18(72)	26(63.41)	0.968
M ^**^	39(41.05)	31(32.63)	25(26.32)	23(58.97)	17(54.83)	17(68)	
L ^***^	33(29.73)	44(39.63)	34(30.64)	21(63.63)	34(77.27)	20(58.82)	
***Eating raw animal products***							
Yes	42(30.66)	58(42.34)	37(27)	25(59.52)	40(68.96)	21(56.75)	0.728
NO	58(35.58)	42(25.77)	63(38.65)	34(58.62)	29(69.04)	42(66.66)	

* H: Living in high-income areas;

** M: Living in middle-income areas;

*** L: Living in low-income areas

## Discussion

The worldwide prevalence of diabetes was estimated at 7.7% in 2030 which represents an increase of about 69% and 20% in developing and developed countries respectively (12). Regarding the possibility of suppressing the immune system in this chronic disease, patients will be at risk for infectious diseases caused by fungal, bacterial, viral and other infectious agents that can involve reducing the quality of life or morbi-mortality of patients with diabetes mellitus (13). However, parasitic diseases in these patients have been less considered.

The association between toxoplasmosis and some other diseases such as chronic disorders and neuropsychiatric disease has been studied previously (17–20). In people with healthy immune systems, toxoplasmosis is usually asymptomatic, which leads to a chronic infection and parasite cysts in body tissues, especially in the brain (5, 9). On the other hand, due to the variety of risk factors, especially immunodeficiency diseases, toxoplasmosis is also particularly important (10, 19).

In the current study, although *T. gondii* seropositivity was higher in diabetic patients, especially in type II diabetes, there was no credible evidence for the correlation between toxoplasmosis and diabetes mellitus based on statistical analysis. Using highly sensitive chemiluminescence immunoassay (CLIA) method, dividing the studied groups into diabetes mellitus type I and II and evaluation of toxoplasmosis among these groups compared with the control group are the strengths of this study. Some other studies have also been designed by other researchers to evaluate *T. gondii* infection in diabetic patients.

The results of a study on 91 diabetic patients and 93 healthy non-diabetic controls by ELISA method, showed a two-fold risk of *T. gondii* infection in diabetic patients compared with non-diabetic individuals (21). The results of this study are inconsistent with the present investigation. This disagreement could be due to differences in the geographical area, demographic characteristics, the prevalence of *T. gondii*, and sensitivity of the laboratory methods. In Iran significantly high rates of anti- *T. gondii* antibodies have been reported in diabetic pregnant women (22). Moreover, in Turkey, more than double rate of infection among patients with type I diabetes compared with healthy individuals (23). Those results are inconsistent with our findings; the main reason for this difference may be due to the patient’s selection criteria. Some other studies similar to our investigation, have not found the association between toxoplasmosis and diabetes. In line with the present study, other studies (24, 25) also did not find a significant difference in toxoplasmosis between diabetic patients and non-diabetic control group.

Although there was no association between toxoplasmosis and type 1 diabetes mellitus *Toxoplasma* infection can be considered as a risk factor for type 2 diabetes. In Iran, the relation between gender, age and raw meat consumption and toxoplasmosis (8); however we have not found such a relation in the present investigation. Nevertheless anti- *T. gondii* IgM cases were more among diabetic female patients and people who eat raw animal products. Although we have not found any evidence for the correlation between *T. gondii* infection and diabetes mellitus but considering health care will be necessary in order to reduce the risk of toxoplasmosis in diabetic patients especially in pregnant women and immunodeficient patients.

## Conclusion

Although the results of this study showed no statistically significant relationship between diabetes and toxoplasmosis but further investigations especially experimental studies using animal models are needed. Studying on other parasitic infections in these patients is inevitable. Furthermore, these findings would not be contrary to the need for healthcare in order to the prevention of infectious disease in diabetic patients.
